# Twelve Antioxidant Peptides From Protein Hydrolysate of Skipjack Tuna (*Katsuwonus pelamis*) Roe Prepared by Flavourzyme: Purification, Sequence Identification, and Activity Evaluation

**DOI:** 10.3389/fnut.2021.813780

**Published:** 2022-01-21

**Authors:** Jiao Wang, Yu-Mei Wang, Long-Yan Li, Chang-Feng Chi, Bin Wang

**Affiliations:** ^1^Zhejiang Provincial Engineering Technology Research Center of Marine Biomedical Products, School of Food and Pharmacy, Zhejiang Ocean University, Zhoushan, China; ^2^National and Provincial Joint Laboratory of Exploration and Utilization of Marine Aquatic Genetic Resources, National Engineering Research Center of Marine Facilities Aquaculture, School of Marine Science and Technology, Zhejiang Ocean University, Zhoushan, China

**Keywords:** skipjack tuna (*Katsuwonus pelamis*), roe, antioxidant activity, antioxidant peptide, oxidative damage, cytoprotection

## Abstract

For using aquatic by-products to manufacture high-value products, Skipjack tuna (*Katsuwonus pelamis*) roes were degreased, pretreated with microwave, and hydrolyzed using five proteases. The protein hydrolysate (TRPH) generated using Flavourzyme displayed the strongest 2,2-diphenyl-1-picrylhydrazyl (DPPH) radical scavenging activity. Twelve antioxidative peptides were prepared from TRPH by ultrafiltration and chromatography methods and determined to be SGE, VDTR, AEM, QDHKA, TVM, QEAE, YEA, VEP, AEHNH, QEP, QAEP, and YVM with molecular weights of 291.24, 489.50, 349.41, 597.59, 349.44, 475.42, 381.36, 343.37, 606.58, 372.35, 443.42, and 411.49 Da, respectively. AEM, QDHKA, YEA, AEHNH, and YVM presented the strongest scavenging activity on DPPH radical (EC_50_ values of 0.250±0.035, 0.279±0.017, 0.233±0.012, 0.334±0.011, and 0.288±0.015 mg/ml, respectively), hydroxyl radical (EC_50_ values of 0.456±0.015, 0.536±0.021, 0.476 ± 0.051, 0.369 ± 0.052, and 0.413 ± 0.019 mg/ml, respectively), and superoxide anion free radical (EC_50_ values of 0.348 ± 0.018, 0.281 ± 0.013, 0.305 ± 0.022, 0.198 ± 0.011, and 0.425 ± 0.021 mg/ml, respectively). Moreover, AEM, QDHKA, YEA, AEHNH, and YVM presented high lipid peroxidation inhibition ability, Ferric-reducing power, and significant protective function on H_2_O_2_-induced Chang liver cells. Therefore, AEM, QDHKA, YEA, AEHNH, and YVM could be natural antioxidant ingredients used in pharmaceutical and functional products.

## Introduction

Tuna and tuna-like species, such as bonito, *Thunnus albacores, T. alalunga*, and *Katsuwonus pelamis*, are favored for their desirable and unique taste, high edible safety, and excellent nutritive value on human health, derived from their rich and high-quality nutrients, such as proteins, unsaturated fatty acid, essential amino acids, and vitamins (1–3). The commercial catches of tuna species exceeded 7.9 million tons, which account for 9% of global total marine catches and make an important contribution to food security and income in developed and developing countries (4, 5). However, exceeding 70% tuna catches, including tuna viscera, head, bone, and skin, are regarded as by-products during the producing cans (3, 6–8). Considering the unusually high quantity of tuna by-products, just like as their environmental effects induced by random disposal and elimination, the large-scale preparation of nutritional ingredients from these by-products is giving rise to sustained concerns (3, 9). In addition, these by-products are tried for the development of fish bait, fish oil-enriched DHA, fertilizer, and pet food and feed according to their different nutrient compositions (3, 10). However, these applications still do not achieve high-value utilization of tuna resources, and it is urgent to make rational and effective use of these by-products (10, 11).

Protein hydrolysates and bioactive peptides (BPs) are potential functional ingredients for the formulation of health products because their excellent properties, such as easy absorption, high nutrition, and significant biological activities (10, 12, 13). At present, active protein hydrolysates and BPs derived from different fishes and their processing by-products have been prepared using enzymatic hydrolysis technology (10, 14, 15). In addition, oxidative stress induced by toxic peroxy radicals can lead to cell malfunction, which has been strongly close conjunction with human aging and chronic maladies (16, 17). Antioxidant peptides (APs) draw great interest because of their potential application in treating chronic ailments, for example, inflammation, neurodegenerative disorders, immunopathy, diabetes mellitus, and Parkinson's and Alzheimer's diseases (18–20). For example, Pro-Gly-Tyr (PGY) could scavenge intracellular reactive oxygen species (ROS) through upregulating the expression abilities of catalase (CAT) and superoxide dismutase 1 (SOD1) in 2,2'-azobis-(2-amidinopropane dihydrochloride) (AAPH)-induced HepG2 cell (21). FPYLRH could defend DNA from ROS injury, raise the levels of SOD and GSH-Px, and cut down the generation quantities of nitric oxide (NO), ROS, and malondialdehyde (MDA) in H_2_O_2_-injured HUVECs (22). Hexapeptides of WAFAPA and MYPGLA revealed greater protecting activity than carnosine against Fenton's reagent-induced oxidative damage of DNA and proteins (23). GGFDMG could decrease the ROS-mediated intracellular oxidative injury of RAW 264.7 through increasing the levels of intracellular antioxidative enzymes to clear away the ROS (24). AAVPSGASTGIYEALELR could activate the DAF-16 signaling pathway to lower the oxidative damage of organisms (25). Therefore, APs can activate the intracellular antioxidant system to increase the activity of antioxidant enzymes, which can effectively scavenge ROS and reduce the damage caused by oxidative stress (19, 22).

Skipjack tuna (*Katsuwonus pelamis*) is the main resource of canned fish and shares of more than 70% of tuna catches (3, 26, 27). Many researches have utilized by-products of skipjack tuna to produce some functional components, such as collagens and gelatins (28, 29), BPs (9, 21, 30), oil and lipid (11, 31), and multiple sulfated polysaccharides (32). Roe is one of the main by-products of skipjack tuna and is rich in crude proteins with the content of 21.00 ± 0.06% of tuna roe (on a wet weight basis) (33). To make full use of by-products to develop high-value products, APs were isolated from protein hydrolysate of skipjack tuna roes in the study. In addition, the radical scavenging activity, lipid peroxidation inhibiting capability, and cytoprotection on H_2_O_2_-induced damage of Chang liver cells of the isolated APs were evaluated systematically.

## Materials and Methods

### Materials and Reagents

Skipjack tuna roes were offered friendly by Ningbo JinRi Food Co., Ltd. (China). Chang liver cells were bought from Cell Bank of the Chinese Academy of Sciences (Shanghai, China). 2,2-diphenyl-1-picrylhydrazyl (DPPH), pepsin, papain, trypsin-EDTA, trypsin, and 3-[4,5-dimethylthiazol-2-yl]-2,5 diphenyl tetrazolium bromide (MTT) were purchased from Sigma-Aldrich (Shanghai) Trading Co., Ltd. (China). Flavourzyme and Alcalase were purchased from Novozymes Biotechnology Co., Ltd. (Tianjin, China). DEAE-52, RPMI modified medium (RPMI-1640), Sephadex G-15, and penicillin–streptomycin solution were purchased from Shanghai Yuanju Bio-Tech Co., Ltd. (Shanghai, China). APs of TRP1 to TRP12 with purity higher than 98% were synthesized in Apeptide Co. Ltd. (Shanghai, China).

### Preparation of Protein Hydrolysate From Skipjack Tuna (*K. pelamis*) Roes

The skipjack tuna roes were thaw, broken by the high-speed homogenizer, and degreased using the method described by Zhao et al. (25). In brief, isopropanol was added into the roe homogenate with a liquid–solid ratio of 4:1 (v/w), and the mixed solution was homogenized and stood at 4°C for 60 min. After that, the mixed solution was centrifuged at 6,000 g for 20 min, and the residue was defatted using isopropanol for three times. Finally, the defatted roe residue was dried using incubator at 35°C.

### Screening of Protease Species

Defatted roe residue was suspended in deionized water to prepare the 10 % (w/v) protein slurry. After that, the mixed solution was treated with microwave at 420 W for 147 s and hydrolyzed for 7 h separately using trypsin at pH 8.0, 37.0°C, Flavourzyme at pH 7.5, 45°C, papain at pH 7.0, 50°C pepsin at pH 2.0, 37.0°C, and Alcalase at pH 9.0, 50°C, with 2.0% (w/w) protease dose. The hydrolysate solutions were heated at 95°C water bath for 10 min to inactivate proteases, centrifuged at 12,000 g for 15 min, and lyophilized. The antioxidant activities of the prepared protein hydrolysates were evaluated using DPPH radical (DPPH•) scavenging assay, which was described by Li et al. (34). Then, the protein hydrolysates generated using Flavourzyme showed the strongest DPPH• scavenging activity among five protein hydrolysates.

### Optimizing the Hydrolysis Parameters of Flavourzyme

For getting high antioxidant activity hydrolysate from skipjack tuna roes, an orthogonal L_9_(3^3^) experiment was designed and employed to optimize the hydrolysis parameters of Flavourzyme, including solid–liquid ratio (1:6, 1:8, 1:10, 1:12, and 1:14), pH value (6.0, 6.5, 7.0, 7.5, and 8.0), and hydrolysis time (4, 5, 6, 7, and 8 h) (Table 1). Finally, tuna roe protein hydrolysate prepared by Flavourzyme hydrolysis under optimum condition was referred as TRPH and used for the following experiments.

**Table 1:** Orthogonal array design matrix L_9_(3^3^) and results for optimizing the hydrolysis conditions of Flavourzyme.

**No**.	**Factor**			**DPPH·scavenging activity (%)**
	**A (solid–liquid ratio)**	**B (time/h)**	**C (pH)**	
1	1:8	6	7	35.22
2	1:8	7	7.5	61.13
3	1:8	8	8	39.51
4	1:10	6	7.5	59.64
5	1:10	7	8	51.31
6	1:10	8	7	34.92
7	1:12	6	8	41.96
8	1:12	7	7	37.66
9	1:12	8	7.5	55.79
K1	45.287	45.607	35.933	
K2	48.623	50.033	58.853	
K3	45.137	43.407	44.260	
R	3.486	6.626	22.920	

### Purification of APs From TRPH

**Figure 1:**
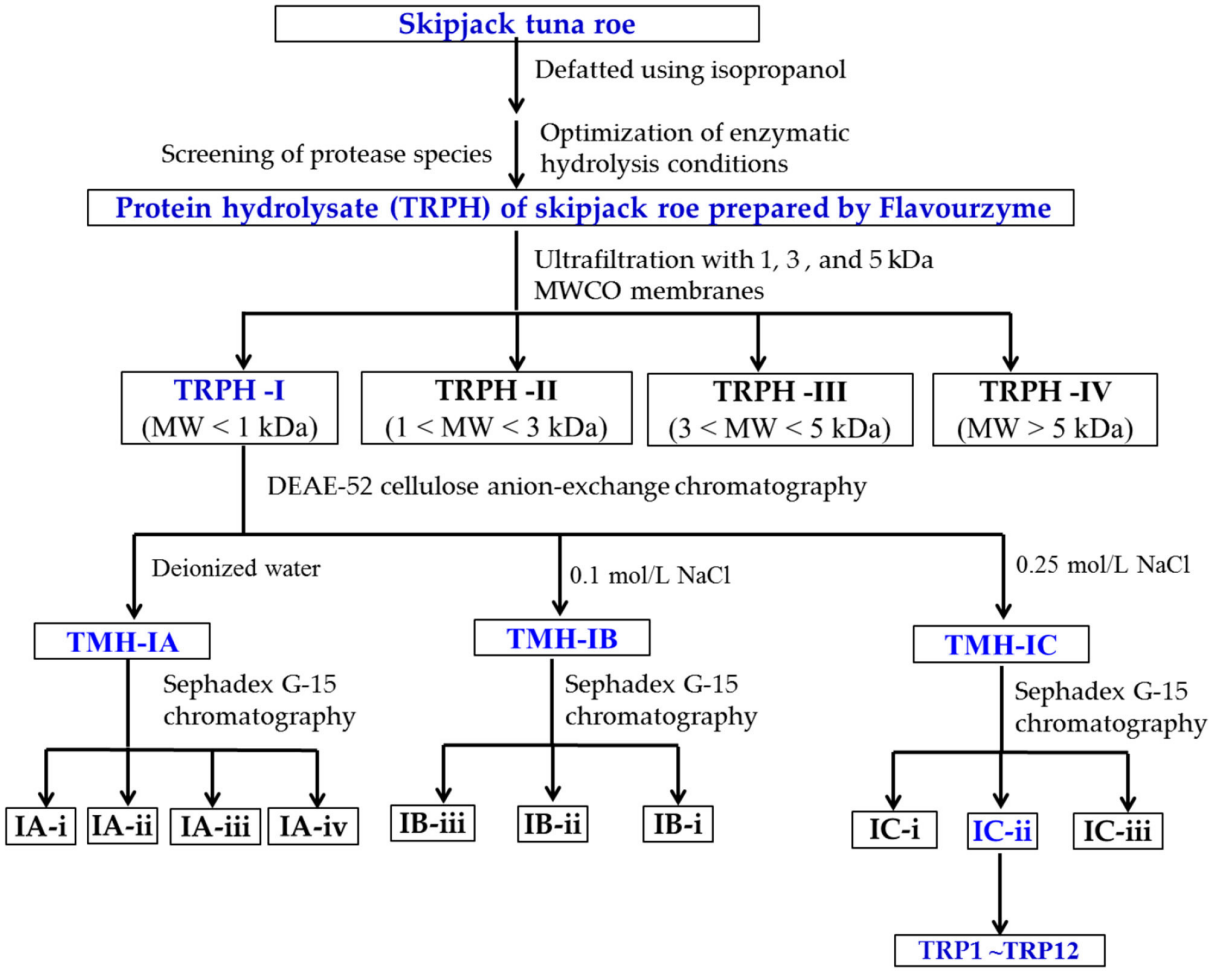
Flow diagram of isolating antioxidant peptides (APs) from protein hydrolysate (TRPH) of skipjack tuna (*Katsuwonus pelamis*) roe prepared by Flavourzyme.

Antioxidant peptides were purified from TRPH using the following designed process (Figure 1).

TRPH solution (50.0 mg/ml) was first divided into different components using molecular weight (MW) cutoff ultrafiltration membranes of 1, 3, and 5 kDa, respectively. Four resulting components, defined as TRPH-I (MW < 1 kDa), TRPH-II (1 < MW < 3 kDa), TRPH-III (3 < MW < 5 kDa), and TRPH-IV (MW > 5 kDa), were collected, dialyzed with MW cutoff dialysis tube of 100 Da, concentrated under reduced pressure, freeze-dried, and detected their DPPH• scavenging activity.

In total, 6 mL of TRPH-I (50.0 mg/ml) was added to the DEAE-52 cellulose column (3.8 × 150 cm) pretreated by deionized water, and the column was eluted using 300 ml deionized water and two kinds of NaCl solutions (0.10 and 0.25 mol/L) in succession at a flow rate of 1.0 ml/min. Then, three fractions (TRPH-IA to TRPH-IC) were washed out from the column, collected according to the chromatogram at 280 nm, desalted using 100 Da dialysis tube, and freeze-dried.

In total, 5 ml of TRPH-IA, TRPH-IB, and TRPH-IC solutions (50.0 mg/ml) were separately injected into the chromatography column of Sephadex G-15 (2.0 × 120 cm) and washed out using deionized water with a flow rate of 0.6 ml/min. The resulted eluent was collected each 2 min and detected at 280 nm. Finally, nine peptide components named IA-I, IA-ii, IA-iii, IB-I, IB-ii, IB-iii, IC-I, IC-ii, and IC-iii were enriched in accordance with the chromatogram maps of TRPH-IA, TRPH-IB, and TRPH-IC, respectively. The prepared peptide fractions were desalted using 100 Da dialysis tube and freeze-dried.

IC-ii (20 μl, 100.0 μg/ml) with the highest DPPH• scavenging activity was pretreated with a microporous membrane (0.22 μm) and subsequently isolated by an HPLC column of Eclips XDB-C18 (4.6 × 250 mm, 5 μm) in Agilent 1200 system, and the Eclips XDB-C18 column was washed out using a gradient of acetonitrile containing 0.05% trifluoroacetic acid (1% acetonitrile in 0 to 4 min; 1–11% acetonitrile in 4–17 mins; 11%−100% acetonitrile in 17–20 mins; 100% acetonitrile in 20–25 mins). The elution velocity of the mobile phase is 0.8 ml/min, and the elution solution was monitored at 280 nm. According to the chromatographic peaks, 12 peptides (TRP1 to TRP12) were purified from IC-ii.

### Analysis of Sequences and MWs of 12 APs (TRP1 to TRP12)

The N-terminal amino acid sequence of 12 APs (TRP1 to TRP12) was determined by the Edman degradation method using an Applied Biosystems 494 protein sequencer (Foster City, CA, USA). MWs of 12 APs (TRP1 to TRP12) were measured by a Q-TOF mass spectrometer (MS) coupled to an electrospray ionization (ESI) source.

### Radical Scavenging, Lipid Peroxidation Inhibition and Ferric-Reducing Power Assays

The scavenging assays of DPPH•, hydroxyl radical (HO•), and superoxide anion free radical (O^−^_2_•) were performed on the methods described by Wang et al. (18) and Li et al. (34). The half clearance concentration (EC_50_ value) was defined as the AP concentration leading to a fifty per cent decline of the initial radical concentration.

Lipid peroxidation inhibition and Ferric-reducing power assays were performed in accordance with the methods reported by He et al. (35).

### Cytoprotection of 12 APs (TRP1 to TRP12) on H_2_O_2_-Induced Chang Liver Cells

#### Effects of 12 APs (TRP1 to TRP12) on the Cell Viability

The Chang liver cells were cultured according to the described method by Wang et al. (18). In short, Chang liver cells with the density of 1.5 × 10^5^ cells/well were seeded into a 96-well plate, which contained 180 μl of culture media.

After incubation for 24 h, 20 μl of AP solution dissolved in the DMEM medium was joined in the peptide group, and the final concentration reached to 200 μmol/L. In addition, the AP was substituted by phosphate buffer solution (PBS, pH 7.2) in the control group. After incubation for 24 h, MTT solution (20 μl) was added in the control and sample groups and incubate for 4 h. Then, the absorbance value at 570 nm (OD_570nm_) of all groups was measured, and the cell viability was calculated according to the following formula:

Cell viability (%) = (OD_sample_/OD_control_)×100.

#### Protection on H_2_O_2_-Induced Chang Liver Cells

The cytoprotective assay was carried out according to the described method by Wang et al. (18). In brief, the selected Chang liver cells (1.5 × 10^5^ cells/well) were cultured in a 96-well plate and cultured for 24 h. Subsequently, the supernatant of cultured cells was aspirated, 20 μl of APs (TRP1 to TRP12) was added into the protection groups, and the final concentrations of APs came up to 200 μmol/L, respectively. Then, the Chang liver cells were incubated with APs for 8 h, APs were cleared away, and H_2_O_2_ was added into the damage and protection groups and achieved the final concentration of 300 μmol/ml. After that, the cells were cultured for 24 h and determined the OD_570nm_ on the previous method. The positive control group used ascorbic acid solution instead of peptide solution.

### Statistical Analysis

Data are presented as mean ± standard deviation (SD) (*n* = 3). The one-way analysis of variance was applied to compare and analyze the average of each treatment, and the Duncan's multiple range test was used to analyze the significant differences in different groups (*p* < 0.05) (SPSS 20.0 software).

## Results and Discussion

### Optimization of the Preparing Conditions of Protein Hydrolysate of Skipjack Tuna Roes Screening of the Types of Proteases

**Figure 2:**
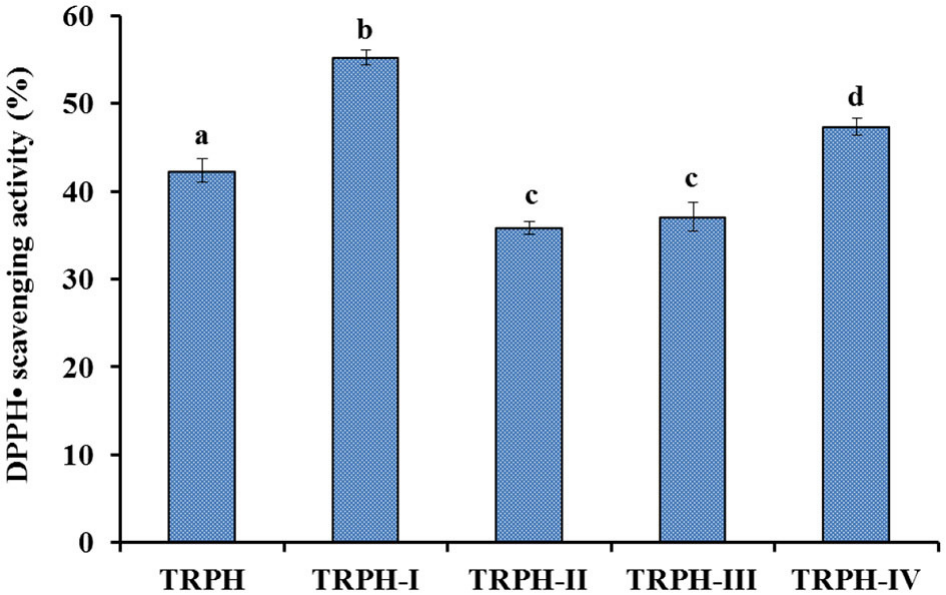
DPPH• scavenging activity (%) of TRPH and its four fractions (TRPH-I to TRPH-IV). All values are means ± SD (*n* = 3). ^a−*d*^Values with same letters indicate no significant difference (*p* > 0.05).

To evaluate the effects of different types of proteases on DPPH• scavenging activity of tuna roe hydrolysates, five kinds of proteases were employed to hydrolyze tuna roe proteins, and the results are shown in Figure 2. At 10.0 mg/ml, DPPH• scavenging activity of protein hydrolysate produced using Flavourzyme was 43.65 ± 1.27%, which was prominently stronger than the activities of the protein hydrolysates produced using trypsin (25.65 ± 0.1.38%), pepsin (31.56 ± 1.65%), papain (35.26 ± 1.26%), and Alcalase (30.58 ± 1.62%), respectively (*p* < 0.05).

Protein hydrolysates derived from food resources show remarkable functions in promoting human health far more than their well accepted nutritional properties (36, 37). These biological functions are in close contact with the structure of biopeptides, which are encrypted within protein sequences and usually are released using enzymatic hydrolysis method (10, 12, 38). Treatment of proteins with different types of proteases can produce several types of protein hydrolysates (9, 35). Therefore, proteases including Alcalase (1, 20, 24, 39), Flavourzyme (3), neutrase (9, 40), pepsin (18), papain (35), trypsin (25), thermolysin (41), and their combinations (6, 7, 13, 25) are frequently used to produce APs from marine organisms and their by-products (10). In Figure 2, protein hydrolysate of skipjack tuna roes produced using Flavourzyme displayed the strongest DPPH• scavenging activity among five hydrolysates, which was in line with the previous literature that the specificity of the proteases could significantly influence the peptide composition of hydrolysates, and those properties are closely related to the biological functions of peptides (12, 42). Therefore, the tuna roe hydrolysate produced using Flavourzyme was named as TRPH and selected for the purification of APs.

### Optimizing the Hydrolysis Parameters of Flavourzyme

According to our preliminary experiment, the influences of hydrolysis parameters including solid–liquid ratio, pH, and hydrolysis time on DPPH• scavenging activity of tuna roe hydrolysates were selected to optimize using an orthogonal L_9_(3^3^) experiment (Table 1). According to the R values, the factors influencing DPPH• scavenging activity of TRPH were listed below using a decreasing order: C > B > A. Therefore, hydrolysis time was confirmed as the most key parameter influencing the DPPH• scavenging activity of TRPH, and the optimal combination of enzymatic hydrolysis parameters was A2B2C2, which was to say, the optimum parameters of Flavourzyme were solid–liquid ratio 1:10, hydrolysis time 7 h, and pH 7.5. At 10 mg/ml, the maximum DPPH• scavenging activity of TRPH was 50.63 ± 0.73% on the optimal parameter combination. Enzymatic parameters employed for the hydrolysis reaction play an indispensable role in the hydrolysis process of proteases (10, 34). Under the optimal enzymolysis level, DPPH• scavenging activity of ethanol-soluble protein hydrolysate of *Sphyrna lewini* muscle could reach 21.76 ± 0.42% at concentration of 10 mg/ml, which was significantly higher than other combination of enzymatic hydrolysis conditions (43). Sila and Bougatef reported that enzymatic conditions could greatly influence the degree of hydrolysis of the protein substrate and the chain length and amino acid composition of peptides, which further modulate their biological activity (10, 44). Then, optimization of those parameters is conducive to hydrolysis efficiency.

### Purification of APs From TRPH by Ultrafiltration

In terms of MW, TRPH was divided into four resulting components using ultrafiltration membranes and defined as TRPH-I, TRPH-II, TRPH-III, and TRPH-IV, respectively. Figure 2 illustrated that DPPH• scavenging activity of TRPH-I was 55.23 ± 0.8% at 6 mg/ml, which was markedly stronger than the activities of TRPH (42.35 ± 1.37%), TRPH-II (35.81 ± 0.76%), TRPH-III (37.09 ± 1.66%), and TRPH-IV (47.38 ± 0.95%) (*p* < 0.05). Li et al. (34) and Chi et al. (44) confirmed that the average MW negatively influenced the antioxidative ability of protein hydrolysates. The present data are in line with the literature that low MW fractions of protein hydrolysates from tuna dark muscle (9), skate cartilage (13), Antarctic krill (18), and miiuy croaker muscle (35) had the highest antioxidant activities. Therefore, TRPH-I with small MW revealed strong DPPH• scavenging ability was selected for further separation.

### Anion-Exchange and Gel Filtration Chromatography

**Figure 3:**
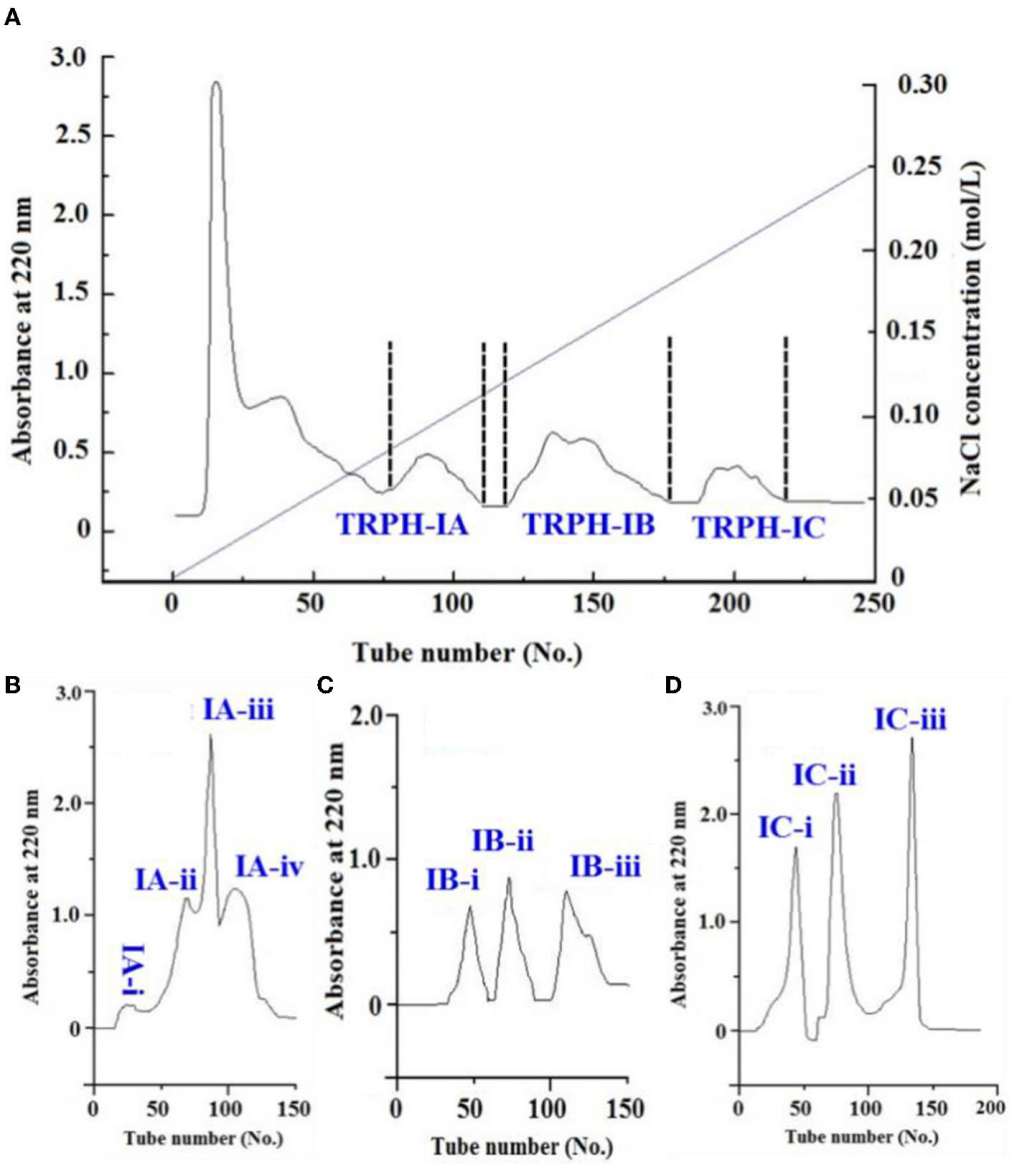
Separation of TRPH-1 by DEAE-52 cellulose anion-exchange chromatography **(A)** and Sephadex G-25 gel filtration chromatography **(B–D)**.

Using an anion-exchange chromatography of DEAE-52 cellulose, TRPH-I (MW < 1 kDa) was fractionated into three fractions, including TRPH-IA, TRPH-IB, and TRPH-IC (Figure 3A). Peptides with basic and/or hydrophobic amino acid residues including His, Lys, and Pro are proved to have strong antioxidative activities (10, 13), basic and/or acidic amino acid residues in peptides can be adsorbed to anion and/or cation exchange resins, and the interaction strength is influenced by the number and location of the charges (45). Therefore, anion-exchange resin of DEAE-52 cellulose has been used to isolate APs from protein hydrolysates (13, 35).

**Figure 4:**
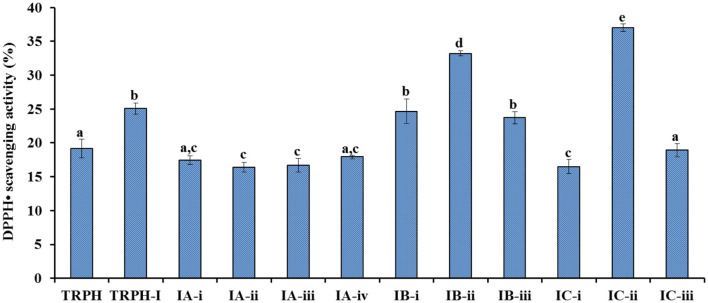
DPPH• scavenging activity of TRPH subfractions using Sephadex G-15 gel filtration chromatography. All values are means ± SD (*n*= 3). ^a−*e*^Values with same letters indicate no significant difference (*p* > 0.05).

Employed a Sephadex G-15 column, four peptide subfractions (IA-i to IA-iv) were isolated from TRPH-IA according to the molecule size of the containing APs (Figure 3B). Then, three peptide subfractions (IB-i to IB-iii) were isolated from TRPH-IB (Figure 3C), and three peptide subfractions (IC-i to IC-iii) were isolated from TRPH-IC3 (Figure 3D). Figure 4 indicated that DPPH• scavenging activity of IC-ii was 37.02 ± 0.54% at 2 mg/ml, which was significantly higher than those of TRPH, TRPH-1, and other nine subfractions (*p* < 0.05). Molecular size is one of the key factors effecting the antioxidative ability of peptides (10, 34, 46). Gel filtration is the most frequently used method for separating BPs with different molecular dimensions from protein hydrolysates (10, 45). The DPPH• scavenging activity of IC-ii was higher than IC-iii, but its MW was higher than that of IC-iii (*p* < 0.05). The finding indicated that the activity of APs is not affected by MW, but by other factors, such as amino acid composition and sequence (10). Then, IC-ii was selected for further RP-HPLC purification.

### RP-HPLC Separation of IC-Ii

**Figure 5:**
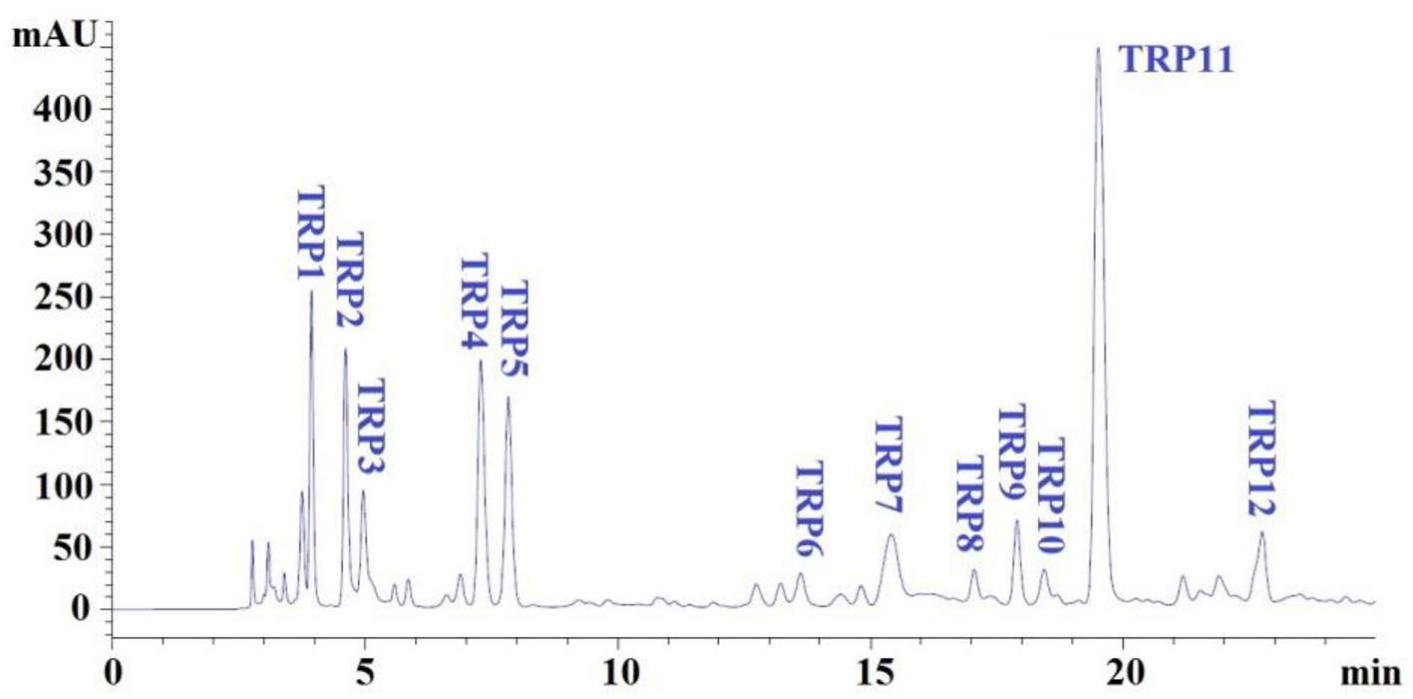
Elution profiles of subfraction IC-ii by RP-HPLC using a gradient of acetonitrile containing 0.05% trifluoroacetic acid at 280 nm.

Subfraction of IC-ii with strong DPPH• scavenging activity was finally isolated by RP-HPLC and its peptide profile is presented in Figure 5. Twelve peptide peaks with retention time (RT) of 3.943 mins (TRP1), 4.617 mins (TRP2), 4.968 mins (TRP3), 7.292 mins (TRP4), 7.837 mins (TRP5), 13.645 mins (TRP6), 15.412 mins (TRP7), 17.105 mins (TRP8), 17.896 mins (TRP9), 18.434 mins (TRP10), 19.507 mins (TRP11), and 22.826 mins (TRP12), respectively, were purified from IC-ii (Table 2). The hydrophobic and hydrophilic properties of peptides play a key role in their RT on an RP-HPLC column, and the ratio of non-polar (methanol, acetonitrile) and polar (water) solvents can adjust the RT (13, 47). Therefore, RP-HPLC is the popular method to purify APs from protein hydrolysates of marine by-products, such as tuna dark muscle (9), skate cartilage (13), Japanese flounder skin (24), and bluefin leatherjacket heads (45). Then, 12 peptides (TRP1 to TRP12) were enriched in large quantities using RP-HPLC and lyophilized for analyzing their purity, structures, and antioxidative activities.

**Table 2:** Retention time, purity, amino acid sequences, and molecular weights of 12 isolated APs (TRP1 to TRP12) from protein hydrolysate (TRPH) of skipjack tuna (*Katsuwonus pelamis*) roe prepared by Flavourzyme.

	**Retention time (time)**	**Purity (%)**	**Amino acid sequence**	**Theoretical mass/observed mass (Da)**
TRP1	3.943	92.54	SGE	291.26/291.24
TRP2	4.617	92.71	VDTR	489.52/489.50
TRP3	4.968	93.53	AEM	349.40/349.41
TRP4	7.292	95.67	QDHKA	597.62/597.59
TRP5	7.837	95.60	TVM	349.45/349.44
TRP6	13.645	93.20	QEAE	475.45/475.42
TRP7	15.412	93.97	YEA	381.38/381.36
TRP8	17.105	95.20	VEP	343.38/343.37
TRP9	17.896	94.76	AEHNH	606.59/606.58
TRP10	18.434	93.85	QEP	372.37/372.35
TRP11	19.507	95.86	QAEP	443.45/443.42
TRP12	22.826	91.35	YVM	411.52/411.49

### Determination of Amino Acid Sequences of 12 Isolated APs (TRP1 to TRP12)

The amino acid sequences and MWs of 12 isolated APs (TRP1 to TRP12) were determined by protein sequencer and ESI/MS, and the data are displayed in Table 2. The sequences of TRP1 to TRP12 were identified as Ser-Gly-Glu (SGE, TRP1), Val-Asp-Thr-Arg (VDTR, TRP2), Ala-Glu-Met (AEM, TRP3), Gln-Asp-His-Lys-Ala (QDHKA, TRP4), Thr-Val-Met (TVM, TRP5), Gln-Glu-Ala-Glu (QEAE, TRP6), Tyr-Glu-Ala (YEA, TRP7), Val-Glu-Pro (VEP, TRP8), Ala-Glu-His-Asn-His (AEHNH, TRP9), Gln-Glu-Pro (QEP, TRP10), Gln-Ala-Glu-Pro (QAEP, TRP11), and Tyr-Val-Met (YVM, TRP12) with MWs of 291.24, 489.50, 349.41, 597.59, 349.44, 475.42, 381.36, 343.37, 606.58, 372.35, 443.42, and 411.49 Da, respectively, and their determined MWs were in line with their theoretical masses (Table 2).

Molecular weight is one of the most important factors impacting the antioxidative abilities of APs (48, 49). APs with smaller size can effectually keep back the lipid peroxidation chain reactions because they are more easily bind to free radicals (10, 39). Moreover, molecular size is a vital restrictive condition for APs stepping over the blood–brain barrier to give play to their pharmacological effects (12, 50). In the study, 12 isolated APs (TRP1 to TRP12) with MWs ranged from 291.26 to 606.59 Da belong to tripeptides, tetrapeptides, and pentapeptides, respectively, which can help them easily get close to the target radicals to exert their antioxidative functions.

### Antioxidant Activity of 12 Isolated APs (TRP1 to TRP12)

To evaluate the antioxidative abilities of 12 isolated APs (TRP1 to TRP12), radical scavenging assay, Ferric-reducing power, lipid peroxidation inhibition assay, and cytoprotection on H_2_O_2_-damaged Chang live cells were conducted.

### Radical Scavenging Activity

According to the EC_50_ values in Table 3, TRP7 (EC_50_ value of 0.233 ± 0.012 mg/ml) showed the highest DPPH• scavenging activity among 12 isolated APs (TRP1 to TRP12), and other peptides were listed on DPPH• scavenging activity in decreasing order as followed by TRP3 (EC_50_ value of 0.250 ± 0.035 mg/ml), TRP4 (EC_50_ value of 0.279 ± 0.017 mg/ml), TRP12 (EC_50_ value of 0.288 ± 0.015 mg/ml), and TRP9 (EC_50_ value of 0.334 ± 0.011). At present, APs were also isolated from other marine protein resources. The EC_50_ values of TRP3, TRP4, TRP7, TRPH8, TRPH9, and TRP12 were less than those of APs from blood clam (TPP: 1.38 mg/ml) (51), Antarctic krill (*Euphausia superba*) (VEKT: 2.23 mg/ml; VEKGK: 3.02 mg/ml; NWDDMRIVAV: 6.19 mg/ml) (18, 19), loach (PSYV: 17.0 mg/ml) (52), skipjack tuna head (ERGPLGPH: 0.93 mg/ml; DAGPYGPI: 1.33 mg/ml; VEE: 3.76 mg/ml) (6), and *Salmo salar* pectoral fin (TTANIEDRR: 2.503 mg/ml) (53). Therefore, TRP3, TRP4, TRP7, TRPH8, TRPH9, and TRP12 could strongly hold back the DPPH• reaction by donating hydrogens or directly clearing away free radicals.

**Table 3:** DPPH•, HO•, and O^−^_2_• scavenging activity of 12 isolated APs (TRP1 to TRP12) from protein hydrolysate (TRPH) of skipjack tuna (*Katsuwonus pelamis*) roe prepared by Flavourzyme.

**Sample**	**Amino acid sequence**	**EC**_**50**_ **(mg/ml)**		
		**DPPH•**	**HO•**	**O^**−**^_**2**_•**
TRP1	SGE	1.342 ± 0.127^a^	2.756 ± 0.118^a^	2.087 ± 0.076^a^
TRP2	VDTR	0.743 ± 0.038^b^	1.521 ± 0.0.083^b^	1.624 ± 0.124^b^
TRP3	AEM	0.25 ± 0.035^c,g^	0.456 ± 0.015^c,d^	0.348 ± 0.018^c,i^
TRP4	QDHKA	0.279 ± 0.017^c,g^	0.536 ± 0.021^d^	0.281 ± 0.013^c,d^
TRP5	TVM	0.537 ± 0.026^d^	0.942 ± 0.0.067^e^	1.069 ± 0.063^e^
TRP6	QEAE	1.766 ± 0.058^e^	3.565 ± 0.127^f^	1.518 ± 0.027^b^
TRP7	YEA	0.233 ± 0.012^c^	0.476 ± 0.0.051^c,d^	0.305 ± 0.022^c,d^
TRP8	VEP	3.313 ± 0.0.047^f^	2.569 ± 0.114^g^	2.876 ± 0.073^f^
TRP9	AEHNH	0.334 ± 0.011^g^	0.369 ± 0.052^c^	0.198 ± 0.011^c,g^
TRP10	QEP	0.972 ± 0.061^h^	2.493 ± 0.095^g^	1.365 ± 0.086^h^
TRP11	QAEP	1.112 ± 0.078^i^	2.095 ± 0.087^h^	2.916 ± 0.112^f^
TRP12	YVM	0.288 ± 0.015^c,g^	0.413 ± 0.019^c,d^	0.425 ± 0.021^i^
Ascorbic acid	–	0.076 ± 0.005^j^	0.182 ± 0.038^i^	0.106 ± 0.009^g^

a−j *Values with the same letters indicate no significant difference (p > 0.05)*.

The EC_50_ values of TRP3, TRP4, TRPH7, TRPH9, and TRP12 on HO• were 0.456 ± 0.015, 0.536 ± 0.021, 0.476 ± 0.051, 0.369 ± 0.052, and 0.413 ± 0.019 mg/ml, respectively, which were notably less than those of other seven isolated APs (*p* < 0.05) (Table 3). In addition, the EC_50_ values of TRP6, TRP7, TRPH8, TRPH9, and TRP17 were less than those of APs from miiuy croaker swim bladders (0.68, 2.31, 2.35, 2.45, and 2.85 mg/ml for FPYLRH, FTGMD, GFYAA, FSGLR, and VPDD, respectively) (50), Antarctic krill (VEKGK: 1.63 mg/ml; SGPA: 2.36 mg/ml; NWDDMRIVAV: 2.61 mg/ml; NGPDPRPSQQ: 2.74 mg/ml) (18, 19), *Misgurnus anguillicaudatus* (PSYV: 2.64 mg/ml) (52), and *Raja porosa* cartilages (IVAGPQ: 5.03 mg/ml) (13). The present finding indicated that the APs from TRPH, especially TRP3, TRP4, TRPH7, TRPH9, and TRP12, could effectively clear HO• to protect biological macromolecules in cells from the oxidative damage induced by HO•.

The EC_50_ values of TRP3, TRP4, TRPH7, TRPH9, and TRP12 on O^−^_2_• were 0.348 ± 0.018, 0.281 ± 0.013, 0.305 ± 0.022, 0.198 ± 0.011, and 0.425 ± 0.021 mg/ml, respectively, which were prominently less than those of other seven isolated APs (*p* < 0.05) (Table 3). Additionally, the EC_50_ values of TRP3, TRP4, TRPH7, TRPH9, and TRP12 were lower than those of APs from monkfish muscle (EDIVCW: 0.94 mg/ml; MEPVW: 0.94 mg/ml) (54), *R. porosa* cartilage (1.61, 1.66, and 1.82 mg/ml for FIMGPY, GPAGDY, and IVAGPQ, respectively) (13), croceine croaker muscle (MILMR: 0.993 mg/ml) (55), Antarctic krill (VEKGK: 1.67 mg/ml; SGPA: 1.86 mg/ml; NWDDMRIVAV: 3.76 mg/ml; NGPDPRPSQQ: 2.03 mg/ml) (18, 19), and muscle (YFLWP: 3.08 mg/ml) and swim bladders (3.04 ± 0.27, 3.61 ± 0.25, 3.03 ± 0.19, 3.35 ± 0.20, and 4.11 ± 0.31 mg/ml for FTGMD, YLPYA, GFYAA, FSGLR, and VPDDD, respectively) of miiuy croaker (35, 50). Therefore, TRP3, TRP4, TRPH7, TRPH9, and TRP12 could clear off the O^−^_2_• damage in cells and biological organs through inhibiting the O^−^_2_• transformed into the highly reactive HO•.

Hydrophobic and aromatic amino acids, such as Ala, Val, Tyr, Met, and His, exert a conclusive role in the bioactivity of APs because those amino acids could assist the interaction between peptide and radical species by heightening the solubility of the peptide in lipids, which further reinforce the antioxidative activities of peptides (10, 23). The imidazole ring structure of His residue is an important active group of APs. In addition, His residue can serve as lipid peroxyl radical trap, hydrogen, and proton donors (56). Saito et al. (57) has proven that His residue is an active amino acid in peptide sequences because tripeptides PHH show the most efficient antioxidative ability among their designed peptides. Therefore, His residue should exert a key role for the antioxidative abilities of TRP4 (QDHKA) and TRP9 (AEHNH). Met residue could turn into a sulfoxide structure to serve as a site of reaction in APs for scavenging free radicals (56). Zhang et al. (6) reported that Met residues were vital factors for the antioxidative activity of EMGPA, WMGPY, and WMFDW. Then, Met residues in TRP3 (AEM) and TRP12 (YVM) were found, indicating that it might act as an active site of TRP3 and TRP12. Tyr residue can act as hydrogen donors to convert radicals into steadier electron conjugated system-phenoxy radicals, which can restrain the radical-mediated propagation of peroxidizing chain reaction (56). So, Tyr residue in TRP7 (YEA) and TRP12 (YVM) could donor hydrogen to stabilize free radical in antioxidant reaction.

Hydrophilic amino acids including Glu/Gln, Asp/Asn, Arg, and Lys also are the key factor for the activity of APs (10, 18). In addition, acidic amino acid residues exert crucial actors in chelating the metal ion and HO· scavenging abilities of APs (58, 59). Memarpoor-Yazdi et al. (60) reported that the basic (Arg) and acidic (Glu and Asp) amino acid residues had significantly positive influences on the antioxidant activities of LDEPDPLI and NTDGSTDYGILQINSR from hen egg white. Glu and Lys residues were important for the Fe^2+^ chelating ability of EVGK and RCLQ (28). Similarly, Glu, Asp, and Lys residues in AEDKKLIQ were conducive to the metal-chelating and radical scavenging activity (61). Therefore, hydrophilic residue in TRP3 (Glu), TRP4 (Gln, Asp, and Lys), TRP7 (Glu), and TRP9 (Glu and Asn) was beneficial to their radical scavenging abilities.

### Ferric-Reducing Power

**Figure 6:**
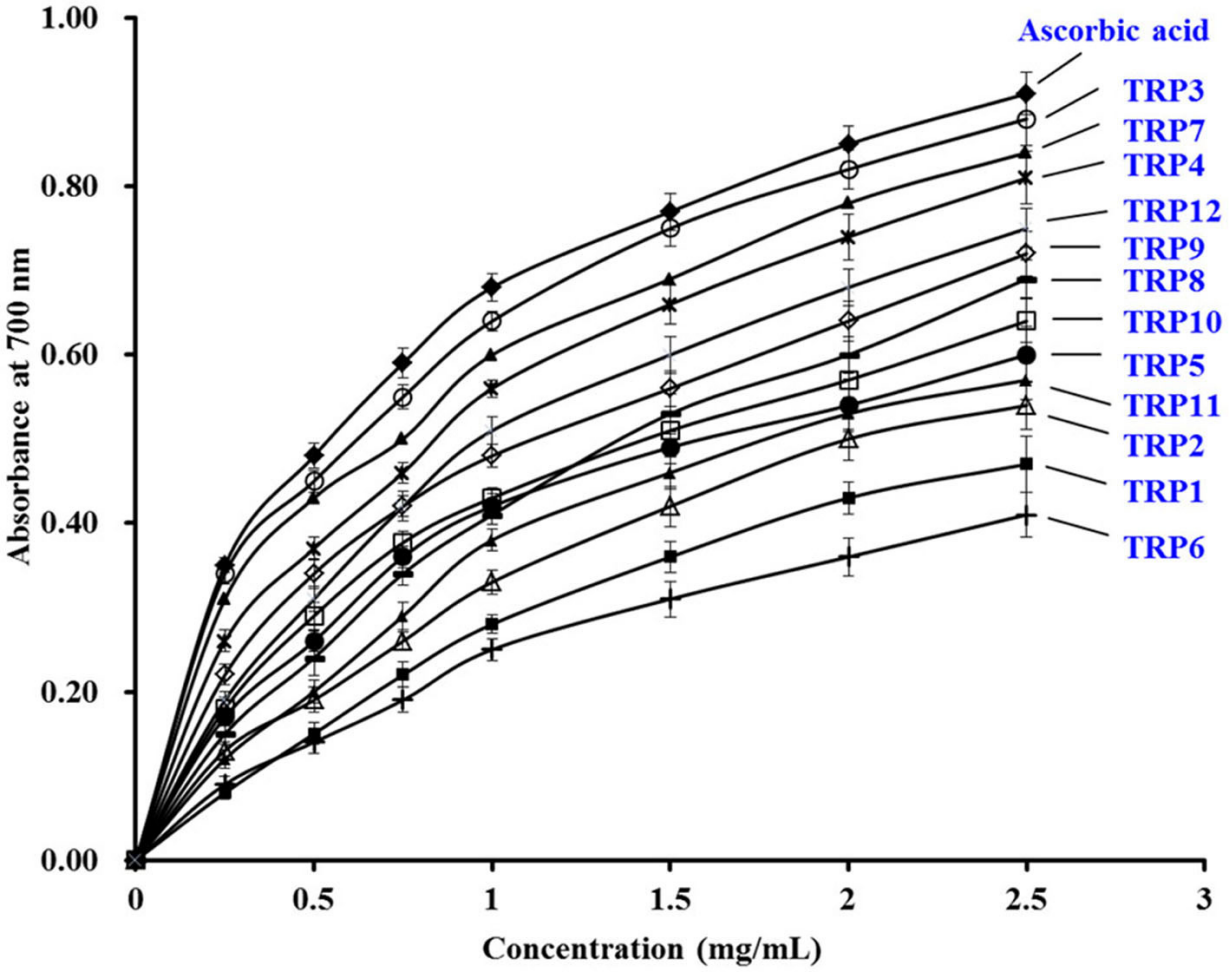
The Ferric-reducing power assays of 12 isolated APs (TRP1 to TRP12) from TRPH. The data are presented as the mean ± SD (*n* = 3).

Figure 6 manifested that the reducing power of 12 isolated APs (TRP1 to TRP12) concentration dependently increased when the AP concentration was progressively increased from 0 to 2.5 mg/ml. In addition, TRP3, TRP4, TRPH7, TRPH9, and TRP12 have higher ability on reducing Fe^3+^/ferricyanide complex into the ferrous form than other seven isolated APs. Nevertheless, the positive control of ascorbic acid presented more reductive power than 12 isolated APs (TRP1 to TRP12). Reducing power gave an index to the ability of antioxidative molecules to donate hydrogen and/or electrons in the reduction reactions of cells, which is a key indicator for evaluating the potential antioxidative activity of antioxidants (6, 34, 35). He et al. reported that FWKVV and FMPLH exhibited the similar reducing power with glutathione (GPS) (35). However, the reducing power of SLPY, QYPPMQY, and EYEA from protein hydrolysate of Antarctic krill was lower than that of GPS (39). Therefore, TRP3, TRP4, TRPH7, TRPH9, and TRP12 could serve as antioxidant additives to provide electrons for reducing the oxidized intermediates in the lipid peroxidation reactions.

### Lipid Peroxidation Inhibition Ability

**Figure 7:**
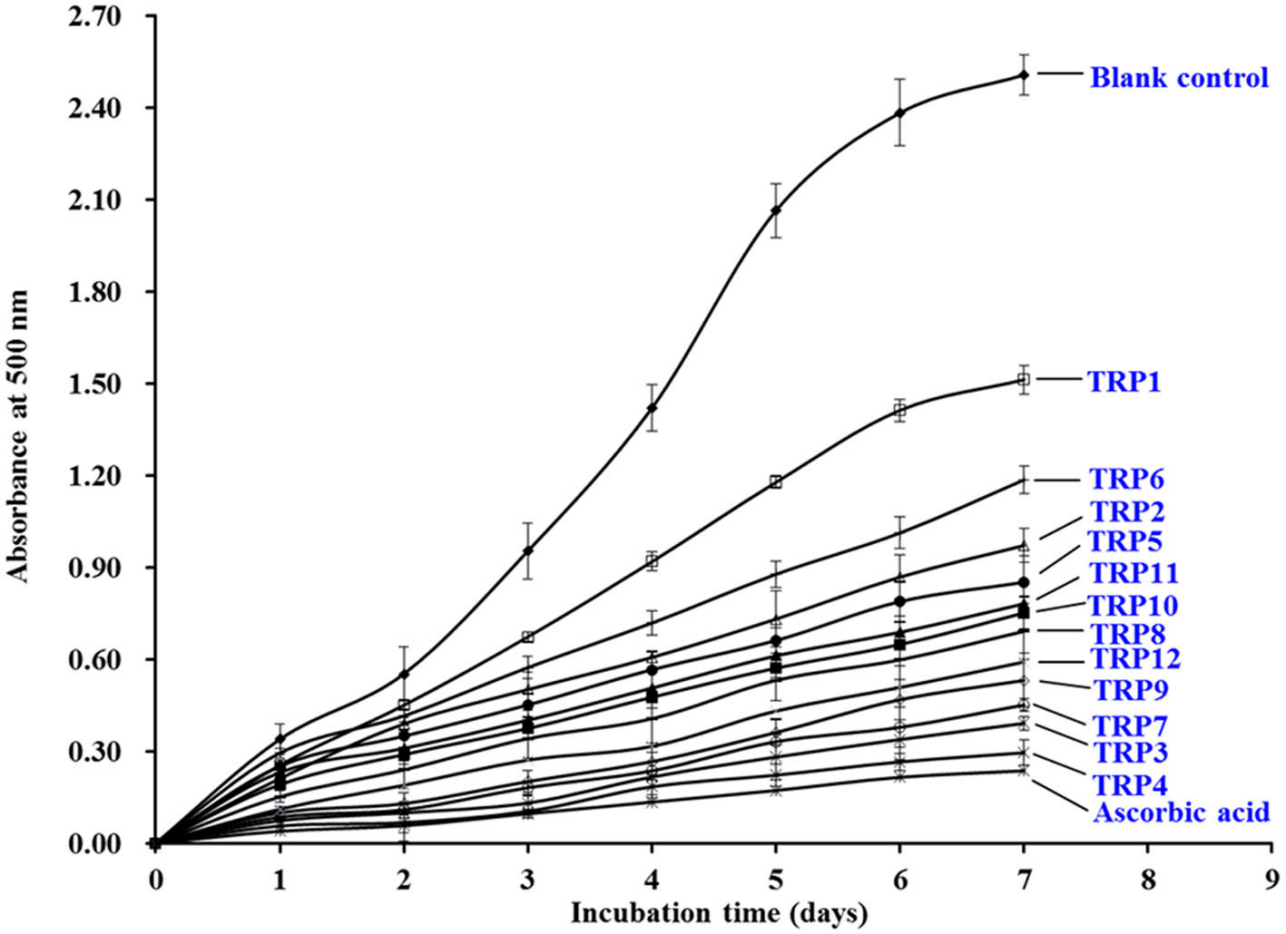
The lipid peroxidation inhibition assays of 12 isolated APs (TRP1 to TRP12) from TRPH. The data are presented as the mean ± SD (*n* = 3).

As displayed in Figure 7, the 500 nm absorbance values of the 12 isolated APs (TRP1 to TRP12) were observably less than that of blank control group when they were incubated at 40°C for 7 days in the experimental system of linoleic acid. Moreover, the inhibiting capabilities of TRP3 and TRP4 got close to the tendency of ascorbic acid (positive control). In Table 3, TRP7 obtained the highest DPPH• scavenging activity, but TRPH9 displayed the strongest HO• and O^−^_2_• scavenging activity. Lipid oxidation is a complicated process and receives the impacts of multiple aspects in food or biological systems. Therefore, lipid peroxidation inhibition assay was employed to synthetically analyze the abilities of the 12 isolated APs (TRP1 to TRP12). The inhibitory activities of TRP3 and TRP4 were similar to those of WMGPY (6), IVAGPQ (13), FMPLH (35), and WDR and PYFNK (36). Previous literatures indicated that the hydrophobic amino acid residues in the sequences of APs are very important for the inhibitory activity of lipid peroxidation (39, 45). Therefore, hydrophobic amino acid residues in TRP3 (Ala and Met) and TRP4 (Ala) could help them easily interact with lipid molecules and scavenge lipid-derived radicals through proton donation.

### Cytoprotective Effects of 12 Isolated APs (TRP1 to TRP12) on H_2_O_2_-Induced Chang Liver Cells

**Figure 8:**
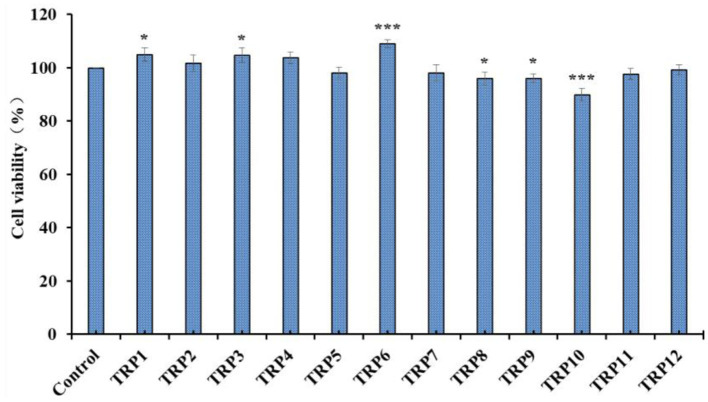
Effects of 12 isolated APs (TRP1 to TRP12) on the viability of Chang liver cells. All data are presented as the mean ± SD (*n* = 3). *p < 0.05 and ***p < 0.001 vs. model group.

Effects of 12 isolated APs (TRP1 to TRP12) on the viability of Chang liver cells at 200 μmol/L were evaluated and the data are shown in Figure 8. The cell viability of TRP10 group was 89.91% ± 2.31%, which was significantly lower than the control and other 11 peptides' groups (*p* < 0.001). The result indicated that TRP6 might have weak cytotoxic effect on Chang live cells. In contrast, the TRP6 group (108.94% ± 1.38%) showed the highest cell viability among all groups (*p* < 0.001). The result indicated that TRP6 could promote the proliferation of Chang live cells. In addition, the cell viability of other 10 peptides groups ranged from 95.93 ± 2.48% (TRP8) to 104.91 ± 2.56% (TRP1), and the cell viability of TRP2, TRP4, TRP5, TRP7, TRP11, and TRP12 and TRP13 groups presented no obvious difference to that of the blank control group (*p* > 0.05). Then, 12 isolated APs (TRP1 to TRP12), except TRP6 and TRP10, could be applied to study the antioxidant-related products.

**Figure 9:**
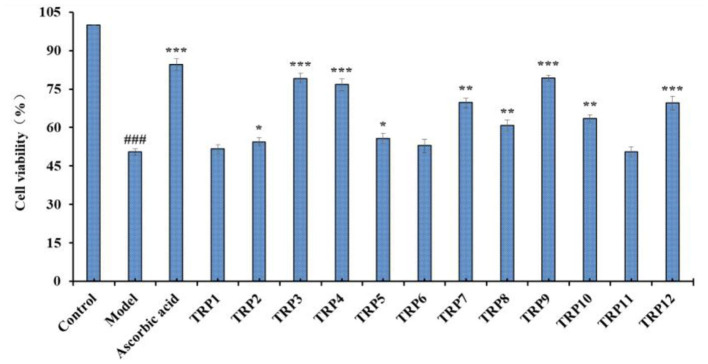
Effects of 12 isolated APs (TRP1 to TRP12) on the cell viability of H_2_O_2_ (300 μmol/L)-induced Chang liver cells. ^###^*p* < 0.01 vs. blank control group; *p < 0.05, **p < 0.01, and ***p < 0.001 vs. model group.

Figure 9 presented the cytoprotective effects of 12 isolated APs (TRP1 to TRP12) on the H_2_O_2_-induced Chang liver cells at the concentration of 200 μmol/L. Compared with the blank control group, the cell viability was remarkably decreased to 50.36 ± 1.27% in model group (*p* < 0.001). However, the cell viability of the positive control (ascorbic acid) group was prominently greater than that of the model group. Additionally, TRP3, TRP4, TRP7, TRP9, and TRP12 showed significant protection functions on the H_2_O_2_-damaged cells with the cell viability of 79.15 ± 1.95%, 76.64 ± 2.33%, 63.63 ± 1.84%, 79.24 ± 1.15%, and 69.52 ± 2.56%, respectively (*p* < 0.001 or *p* < 0.005), but the cell viability of TRP1, TRP6, and TRP11 groups showed no significant difference to the model group. Therefore, TRP3, TRP4, TRP7, TRP9, and TRP12 have high cytoprotective function on H_2_O_2_ damaged Chang liver cells without significant toxicity in this study.

Superfluous ROS can destroy multifarious cellular components during the oxidative stress and further lead to apoptosis due to gene mutation, enzyme inactivation, and membrane potential reduction, which causes chronic maladies (62–64). Bioactive oligopeptides of FWKVV and FMPLH from papain hydrolysate of *Miichthys miiuy* muscle could prominently relieve the oxidative damage of HUVECs triggered by hydrogen peroxide because FWKVV and FMPLH could improve the activity of antioxidative enzymes and bring down the contents of NO, ROS, and MDA (19). KVLPVPEK could activate the Nrf2-ARE pathway in human colon cancer cell (Caco-2 cells), which further induced the overexpression of phase II detoxification enzymes (49). APKGVQGPNG showed the same antioxidant mechanisms in RAW 264.7 cells *via* increasing the level of Nrf2-ARE-mediated heme oxygenase-1 (HO-1) to decrease the levels of NO and ROS (65). Therefore, the available experimental results demonstrated that TRP3, TRP4, TRP7, TRP9, and TRP12 could protect Chang liver cell from H_2_O_2_ damage, and the protective mechanism might be related to activating the Nrf-2 pathway to increase intracellular antioxidative enzymes and antioxidant levels.

## Conclusion

In this study, 12 APs were isolated from protein hydrolysate of skipjack tuna (*K. pelamis*) roes produced using Flavourzyme and identified as SGE, VDTR, AEM, QDHKA, TVM, QEAE, YEA, VEP, AEHNH, QEP, QAEP, and YVM, respectively. Among them, AEM, QDHKA, YEA, AEHNH, and YVM showed the highest radical scavenging activity, Ferric-reducing power, lipid peroxidation inhibition, and protective functions on H_2_O_2_-induced Chang liver cells. Therefore, 12 APs were prepared from skipjack tuna roe, which not only provides technical support for high-value utilization of tuna by-products, but also helps to solve the environmental pollution problem of aquatic processing by-products. More importantly, 12 isolated peptides, especially AEM, QDHKA, YEA, AEHNH, and YVM, might be used as natural ingredients for the development of products with antioxidative functions. In addition, their antioxidant mechanism and physiological function *in vivo* will be systematically evaluated in the follow-up study.

## Data Availability Statement

The original contributions presented in the study are included in the article/supplementary material, further inquiries can be directed to the corresponding authors.

## Author Contributions

JW and L-YL carried out conceptualization, methodology, investigation, data curation, and formal analysis. Y-MW performed data curation, formal analysis, investigation, methodology, supervision, and writing original draft. C-FC and BW involved in conceptualization, funding acquisition, resources, writing, reviewing, and editing. All authors contributed to the article and approved the submitted version.

## Funding

This work was funded by the National Natural Science Foundation of China (No. 82073764) and the Ten-Thousand Talents Plan of Zhejiang Province (No. 2019R52026).

## Conflict of Interest

The authors declare that the research was conducted in the absence of any commercial or financial relationships that could be construed as a potential conflict of interest.

## Publisher's Note

All claims expressed in this article are solely those of the authors and do not necessarily represent those of their affiliated organizations, or those of the publisher, the editors and the reviewers. Any product that may be evaluated in this article, or claim that may be made by its manufacturer, is not guaranteed or endorsed by the publisher.
